# Epistemic fault lines in biomedical and social approaches to HIV prevention

**DOI:** 10.1186/1758-2652-14-S2-S2

**Published:** 2011-09-27

**Authors:** Barry D Adam

**Affiliations:** 1 Department of Sociology, Anthropology and Criminology, University of Windsor, Windsor, Ontario, Canada; 2 Ontario HIV Treatment Network, 1300 Yonge Street #600, Toronto, Ontario, Canada

## Abstract

This paper raises the question of how knowledge creation is organized in the area of HIV prevention and how this concatenation of expertise, resources, at-risk people and viruses shapes the knowledge used to impede the epidemic. It also seeks to trouble the discourses of biomedical pre-eminence in the field of HIV prevention by examining the claim for treatment as prevention, looking at evidence constructed through the biomedical frame and through the lens of the sociology of science. These questions lie within a larger socio-historical context of lagging worldwide attention and funding to prevention in the HIV area and, in particular, neglect of populations at greatest risk. Much contemporary HIV prevention research relies on a population science divided over an epistemic fault line from the communities and individuals who must make sense of the intrusion of a life-threatening disease into their pursuit of pleasure and intimacy. There are, nevertheless, lessons to be learned from prevention success stories among sex workers, injection drug users, and gay and bisexual men. The success stories point to a need for a robust social science agenda that examines: the ways that people are socially organized and networked; the popular strategies and folk wisdoms developed in the face of HIV risk; socio-historical movement of sexual and drug cultures; the dynamics of popular mobilization to advance health; the institutional sources of HIV discourses; and popular understandings of HIV technologies and messages.

## 

*Traditional knowledge handed down from generation to generation helped to save ancient tribes on India’s Andaman and Nicobar Islands from the worst of the tsunami*, *anthropologists say.*

BBC News, 20 January 2005 [1]

*The Bush Administration today announced a plan to expand U.S. tsunami detection and warning capabilities as part of the Global Earth Observation System of Systems* (*GEOSS*), *the international effort to develop a comprehensive*, *sustained and integrated Earth observation system. The plan commits a total of $37.5 million over the next two years.*

Office of Science and Technology Policy

Executive Office of the President,

Washington, D.C.,

14 January 2005

(http://dssresources.com/news/531.php)

Natural cataclysms like tsunamis and epidemics raise critical questions concerning how best to allocate resources to research and action in order to avoid foreseeable recurrences. This pair of news items, following the 26 December 2004 tsunami off the coast of Sumatra that killed 230,000 people in 14 countries, shows contrasting knowledge systems brought to bear in comprehending and constructing the nature of a problem and its response. On one side are decidedly low-tech indigenous knowledge and anthropological research; on the other is capital-intensive, high-tech seismic detection. Perhaps most notable is the almost reflex endorsement of the latter knowledge system by the most powerful government on the planet, and the complex, expensive, global infrastructure put in place to sustain that system.

This small example in the sociology of science can be an occasion for reflecting on the construction and institutionalization of knowledge in the HIV epidemic, in particular the overwhelming belief in, and institutionalization of, a health science of HIV prevention and the marginalization - even at times, erasure - of indigenous knowledges of affected communities and of social science knowledge committed to the documentation and elucidation of local cultures, social contexts and community mobilization.

This paper seeks to trouble the discourses of biomedical pre-eminence in the field of HIV prevention by examining the widely touted claim for treatment as prevention, looking at evidence constructed through the biomedical frame pertaining to this claim and through the lens of the sociology of science. It also examines how policy derived from population-level analysis and biomedical individualism affects people who must manage HIV risk in their everyday lives and social contexts. At the risk of constructing too sharp a contrast between two systems of knowledge, it should be noted at the outset that even in the tsunami example, at least one official body, the (United States) National Academy of Sciences [2], called for “incorporating the latest social science research on hazard education and conducting routine evaluations of education programs” as a key element in a larger strategy of tsunami preparedness. HIV prevention need not be an either/or choice between competing or antagonistic knowledge systems; nor is it just a question of assimilating a highly technicized version of social science as “hazard education and…routine evaluations” into biomedicine.

To echo the language of actor-network theory [3,4], it is rather a question of how community members, scientists and disease entities are networked together into professional systems that assemble, order and institutionalize problems and their solutions. These systems marshal the lion’s share of resources, formulate policy and shape practice intended to impede the advance of the epidemic. The choice to fund some professional systems of knowledge over others by government, industry and scientific funding agencies necessarily has profound consequences on how the challenges of epidemics are met. As such, these organizational paradigms need critical scrutiny to determine what has been included in scientific discourse and what has been relegated to the status of “subjugated knowledges” [5] to determine how well current concatenations of knowledge-producing actors address the questions: (1) how people and resources are best organized to stem the continuing proliferation of HIV infection; and (2) how communities and individual community members are best mobilized to avoid HIV without sacrificing the pursuit of pleasure and intimacy that HIV threatens [6].

## The inversion of prevention priorities

All of these questions lie within a larger socio-historical context of lagging worldwide attention and funding to prevention in the HIV area [7]. The subordination of prevention is no doubt in part a side effect of the need to treat millions who are HIV positive in a world where only a minority of those with HIV in the global south have access to adequate antiretroviral medication. Associated with the perceived imperative to bring treatment to the many in need is a trend to assimilate prevention to the treatment imperative, grounded on the idea that treatment lowers the population-level viral load and thereby lowers the rapidity of HIV transmission [8]. At the same time, treatment as prevention has precedents as a strategy to combat the HIV epidemic as the latest iteration in a history of tensions in the prioritization, integration or takeover of prevention by treatment [9]. Compounding the problem of the small percentage of HIV budgets typically falling to prevention, a disproportionate amount goes to populations who are not at greatest risk.

A recent report examining the percentage of HIV investment of the Global Fund To Fight AIDS, Tuberculosis and Malaria in high-risk populations finds the following: men who have sex with men - US$19 million (2.1% of the US$903 million total); sex workers - US$29 million (3.2%); and people who inject drugs - US$31 million (3.5%). In countries with concentrated epidemics, the report says, funding for HIV prevention interventions targeting most-at-risk populations accounted for 10% of all preventive activities, and in countries with generalized epidemics, for 4% [10]. Even in generalized epidemics, there is clear evidence of much higher rates of HIV infection among most-at-risk populations, such as men who have sex with men, both in the global south and the global north [11-14].

Research investment typically accounts for just a few percentage points of HIV prevention budgets and very often follows a similar pattern of disproportionate funding devoted to populations who are not at greatest risk. For example, an index search of the Canadian Institutes of Health Research Funding Decisions Data website (http://webapps.cihr-irsc.gc.ca/cfdd/db_search?p_language=E&p_competition=EnterCompetitionCodeHere) shows a commitment of $98,830,449 to HIV/AIDS research overall, of which $22,451,362 contain the word “prevention” in funded abstracts, and $9,678,007 also contain “IDU”, $2,351,934 contain “sex work” and $2,168,525 contain “men who have sex with men” In other words, 9.7% of the research budget for grants mentioning HIV prevention also make any mention of men who have sex with men in a country where 51% of the epidemic is concentrated among gay and bisexual men [15]. Finally, investment in prevention research typically goes first to biomedical technologies [16], followed by “intensive practitioner-delivered lifestyle-change interventions” [17], and least, if any, to investigating community mobilization.

Advancement in HIV prevention, as represented in major international policy documents, often appears largely as a question of the development of biomedical technologies or interventions, such as vaccines, microbicides, pre-or post-exposure prophylaxis or circumcision. A perusal of plenaries with a central focus on prevention in the past five international AIDS conferences (Barcelona 2002, Bangkok 2004, Toronto 2006, Mexico City 2008, Vienna 2010) shows 13 of the 21 speakers treating prevention primarily or exclusively as a biomedical technology. The remainder show a familiar pattern of representation of at-risk populations: three speaking on injection drug users, two on women (one of whom looked at sex work), and one on men who have sex with men (MSM). Speakers on HIV prevention in at-risk populations were typically physicians or public health authorities. Social science makes a solo appearance with a psychologist speaking to “conceptual frameworks and HIV/AIDS prevention paradigms” A recent review of the 2010 conference conducted by the Global Forum on MSM and HIV notes that taken together across the conference programme, “only 2.6%, 4.5%, 3.0% and 1.1% of all sessions exclusively focused on MSM, people who use drugs, sex workers and transgender people respectively” [18].

## Troubling the treatment-as-prevention paradigm

The treatment-as-prevention paradigm is grounded on a straightforward proposition: bringing medication to the maximum number of people infected with HIV will not only bring the promise of greatly enhanced survival and quality of life for people living with HIV, but will also greatly reduce their viral loads and the likelihood of passing the virus onto new people. Universal treatment, then, appears to have the potential of having a two-fold effect of saving lives while stalling or even reversing the progress of the epidemic. It is a proposition that makes a good deal of sense from the Olympian viewpoint of population-level planning, professionally directed public policy, and not coincidentally the profitability of the multinational pharmaceutical industry.

Closer examination of this paradigm, even from a strictly biomedical frame of evidence, shows that it comes with a series of qualifications. The evidence for the effectiveness of treatment for prevention is pieced together from a small set of studies on vertical transmission, serodiscordant (presumably monogamous) heterosexual couples [19], ecological studies [20,21], and modelling studies [22]. Equating undetectable viral load with non-infectivity falters with problems of equating viral load results in blood, semen and vaginal secretions [23-25]. In other words, a periodic blood test to determine viral load does not guarantee a similar reading in sexually transmitted fluids. Viral loads turn out, as well, to be somewhat unstable [26,27]. “Blips” that is, periods of elevated viral replication, are not unusual especially at times of activation of other infections, such as sexually transmitted infections. Given the widespread presence and intermittent reactivation of long-term viruses like herpes virus and human papillomavirus in the general population and in at-risk populations in particular, this may not be a rare occurrence. Syphilis and hepatitis C outbreaks in recent years in major cities of North America and Europe [28], particularly among HIV-positive men, point to additional limitations to relying on treatment-related viral reduction as an assurance for non-transmission.

Accomplishing universal treatment may be no mean feat. Taking Canada’s largest province, Ontario, as an example, a context of universal medicare and first-world access to treatment (including many dedicated clinics for HIV treatment), one finds about a third of the HIV-positive population reaching undetectable viral load levels (Table 1). Estimates for the United States appear to be even lower, with 19% of HIV-positive people reaching undetectability levels [29]. Accomplishing widespread viral undetectability, even in highly-resourced settings, then appears to pose major challenges.

**Table 1:** Treatment status of HIV-positive people in Ontario

~9300 HIV+ people do not know they have HIV infection	35%
1700 diagnosed but not in care, i.e. have not had a viral load test	6%
3440 in care but not on ARV	13%
3630 in care, on ARV, and have detectable viral load	14%
8470 have undetectable viral load	32%

Shifting perspective from the population level to that of people who must manage risk in their everyday lives brings quite another range of considerations into view. Recent research done in Australia [30] and the United States shows that “men in 2006 endorsed the prevention treatment beliefs to a greater degree than men in both 1997 and 2005…[and] men who engaged in unprotected anal sex increased their endorsement of these beliefs” [31]. And though belief about treatment effectiveness may influence (un)safe sex practice, unprotected anal intercourse among HIV-positive men does not appear to be associated with actual viral load [32,33]. Furthermore, unprotected anal intercourse appears to be associated with non-adherence to medication [34], a practice very likely to compromise the maintenance of undetectable viral load. Undetectability, in any case, is not the same as the absence of circulating virus; it refers only to the limit of testing capability, which is typically 50 copies per milliliter.

DP Wilson [35] and colleagues estimate that over the course of a relationship of repeated exposure to “undetectable” virus, in a population of 10,000 serodiscordant couples over 10 years, there would still be 215 transmissions from HIV-positive women to HIV-negative men; 425 transmissions from HIV-positive men to HIV-negative women; and 3524 transmissions from HIV-positive men to HIV-negative men. Timothy Hallett [36] and colleagues estimate that “men receiving treatment pose a substantial risk of HIV transmission (22%; 9-37% in uncertainty analysis) to their partners if they do not use condoms”. The male-to-male transmission rate raises questions of just which population is intended by population-level analysis, particularly in epidemics in the global north where men who have sex with men typically account for half of current HIV transmissions.

## Epistemic fault lines: population health and everyday risk

The treatment-as-prevention paradigm, then, deserves some caution even when read from inside a biomedical frame of reference, but perhaps even more problematic is the relationship of the entire paradigm to the larger world of collective risk management. Treatment as prevention and population health science almost always proceed from a series of premises grounded in positivism where practices, characteristics and attributes are abstracted from context and fixed into place as variables, and then correlated through probabilistic statistics. These mathematical manipulations produce a form of actuarial reasoning compatible with the standpoint of state agencies and capitalist enterprises.

The difficulty with this is the fundamental disjuncture between this form of reasoning and the reasoning inherent in navigating risk in everyday life. For example, the insurance industry constructs the category of the high-risk driver as male and under the age of 25 (and penalizes everyone falling into this category with sharply elevated insurance premiums), yet this finding offers little of value to young men, or even to other drivers who encounter young male drivers on the road, on how to drive safely or even reduce driving risk in any way.

In HIV research, actuarial paradigms produce information that is notoriously difficult to translate into prevention practice or advice for those who must cope with HIV risk every day. While probabilistic statistics may be able to identify “significant” differences in risk, based on a spread of a few percentage points on a variable, HIV infection is a binary: you either get it or you do not. Cindy Patton [37] typifies this fault line as one between “witnessing disease” at the population level and “witnessing illness”, arguing, “Because witnessing disease claims its superiority on the basis of population-level viral reduction and cost, treatment-as-prevention programs cannot ‘see’ the individual” as an actor who must manage disease, or risk of disease, in everyday life.

Bringing population-level reasoning to grassroots practice can, at times, produce paradoxical or noxious results. Some recent research [38], for example, shows that younger men who have older partners have higher rates of seroconversion compared with those who do not (a finding precedented by research on age-mixing in African heterosexual transmission and among injection drug users). The finding shows impeccable abstracted positivist logic: A correlates with B, and therefore something should be done. But what? An editorial in the *Journal of Acquired Immune Deficiency Syndromes*[*39*] raises the alarm that public health officials have not yet acknowledged that age mixing can be a significant driver in HIV epidemics”

Of course, this logic can easily be extended outward. Latino men, and even more so, African American men, have higher rates of HIV. Men who have receptive sex have higher rates of HIV. And all of these measures are just proxies for HIV positivity, so obviously people living with HIV have a 100% HIV rate. So the average gay man is to select only young, white, HIV-negative partners who are exclusive tops and all will be well? This kind of reasoning remains resolutely asocial, ahistorical and out of tune with basic human psychology. It has no context, no sense of social interaction, and cares nothing for real risk management. It takes no interest in the ways in which public health advice of this type stokes racism, ageism, homophobia and AIDS phobia, and how the heightening of invidious social distinctions of this kind ultimately contributes to precisely the social dynamics of stigma that shut down disclosure, disempower those on the receiving end of discrimination, and heighten risk [40,41]. Fortunately male desire will never be disciplined by this kind of authoritarian positivism and men will continue to love, care for, and have sex with men across age, sex, race and sero-status lines.

The result is a population science establishment divided over an epistemic fault line from the communities and individuals who must make sense of the intrusion of a life-threatening disease into their pursuit of pleasure and intimacy. While the “social determinants of health” paradigm in health research does at least recognize a world beyond biology that is influential in human health, it still remains firmly ensconced on the “population health” side of the epistemic fault line. It is on this fault line that are built various “knowledge translation and exchange” (KTE) enterprises and “community-based research” (CBR) initiatives where community members are brought in to monitor the apparently nefarious ways of researchers.

At its best, CBR and KTE do engage community members at every stage of the research process, though this engagement may or may not be facilitated by the fundamental logic of the research paradigm. At its worst, KTE becomes a pipeline designed to push through popular resistance in order to reassert the population health paradigm and CBR devolves to AIDS service organizations to act as a communication circuit or buffer between health science and people affected by the epidemic, though they are often not in a position to have the skills or resources to carry out a mandate of this sort. The question that remains is: why can there not be prevention knowledge that starts from the grounded experience of people who deal most directly with HIV risk rather than starting from a population level of analysis?

## Epistemic fault lines: prevention technologies and practices

There can be no doubt that additional effective prevention technology would be most welcome in the realm of HIV prevention. Thirty years since the identification of HIV, the rather low-tech condom remains the primary defence against sexual transmission of HIV, and it is a technology with well-known drawbacks in physical sensation and the expression of intimacy. During that time period, a good deal of research money has been poured into prevention technologies in the treatment-as-prevention strategy and beyond. The problem is, of course, that vaccines, microbicides, pre- and post-exposure prophylaxis, and circumcision have had only very limited success [42]. An effective vaccine still appears to be a long way off. Circumcision may have some impact on the epidemiological numbers, particularly in generalized, largely heterosexual epidemics in populations where circumcision is currently low, though even this claim is not without its critics [43]. It seems likely to have only negligible effect in countries with other epidemic patterns. Recent research in pre-exposure prophylaxis is showing approximately 39% to 44% effectiveness [44,45] and may find a place as a supplement to condom use, but scarcely as a replacement for it.

Perhaps the most striking, but inadvertent, lesson to be drawn from these studies is that all biomedical prevention technologies are also social interventions, whether that is explicitly recognized or not. Pre-exposure prophylaxis, like condom use, is clearly strongly dependent on “adherence” a term often associated with patient recalcitrance and management, but which glosses the very large realm of how interventions fit with everyday exigencies, cross-cutting demands of home and workplace, available options, economic resources and interpretive frameworks of the people who are to adopt these technologies.

There are nevertheless some well-recognized HIV prevention success stories, for example: the Songachi project in India and the 100% Condom Programme in Thailand among sex workers; mobilization among injection drug users and development of needle exchange and safe injection sites; and the mobilization of gay and bisexual men in Europe, North America and Australia in the 1980s and 1990s. All of these examples resulted in major reductions of HIV infection in diverse populations. If there is a common thread running through these examples, it is that the success of relatively low-tech prevention strategies, based on condom use or needle exchange, comes about only through the cooperation and coordination of all relevant stakeholders, from local government, public health and related business sectors through to community organizations and most importantly, to affected populations themselves.

The United Nations report on the Thai 100% Condom Programme concludes that effectiveness was dependent on a “collaborative effort among local authorities, public health officers, sex establishment owners, and sex workers to ensure that clients could not purchase sexual services without condom use in the province”. When the programme was implemented, rates of sexually transmitted infections dropped “quickly and significantly” [46]. (Lack of comparable concentrated effort in addressing the epidemic among men who have sex with men in Thailand has resulted in rising rates [47].)

Reviews of the Songachi project come to a similar conclusion. At the community level, this included: (1) redefining the problem in a way that does not stigmatize individuals; (2) helping the community assume responsibility by highlighting ways in which the short- and long-term benefits of implementing safer acts are apparent both for the individual and the community; (3) reducing environmental barriers to implementation; and (4) providing resources. The group level of change involved building relationships among those in the target population, between sex workers and stakeholders, and between the initial change agents and sex workers, thus building a supportive network to sustain the programme over time. At the individual level, the programme provided information and education, built skills and addressed social perceptions of the sex workers [48].

Drug users succeeded in reducing drug-related harm through small group activities and, in some instances, formal organizations in several locales in the early days of the epidemic [49]. Needle exchange programmes clearly require the cooperation of legal regimes and municipal authorities, including police, public health, outreach workers, and drug-using networks and individuals [50]. Gay communities in the global north were among the first to respond to the AIDS crisis, building on networks developed in recent years of community and movement formation and impelling health establishments and state agencies to overcome inertia, even antipathy [51-55]. The result was a rapid, major reform of sexual behaviour and a sharp drop in HIV infection over the course of a decade.

Perhaps especially notable about these prevention successes is that they typically employ remarkably low-tech solutions among populations that receive slim allocations of restricted prevention budgets. They also pose significant questions to social science concerning the ways in which at-risk populations develop strategies of resistance in the face of the HIV epidemic and how these strategies are best supported by community, state and sometimes business organizations around them. Prevention good-news stories of this kind are clearly just the beginning of an adequate strategy for stemming HIV transmission, but scarcely enough in themselves. Men who have sex with men, for example, despite impressive gains in the first two decades of the epidemic, continue to be infected at rates more than 44 times that of the men around them [56].

## Techno-eschatology, or why there needs be a robust social science agenda

Professional knowledge systems in HIV, then, have invested heavily in biomedical technologies and have privileged particular paradigms in the health sciences, leaving other knowledge networks relatively underdeveloped and under-resourced. Much of the first decades of HIV have been characterized by a certain “techno-eschatology”, that is, a tendency to wait for a definitive answer or historical turning point to be delivered by science and technology. That tendency to keep waiting for deliverance from an epidemic that has already killed 25 million has tended to divert attention away from what has and can be accomplished now.

Impeding the epidemic is work that needs tools available in the social sciences. These include examining how HIV moves (or is slowed) according to: the ways that people are socially organized and networked; the popular strategies and folk wisdoms developed in the face of HIV risk; socio-historical movement of sexual and drug cultures; the dynamics of popular mobilization to advance health; the institutional sources of HIV discourses; and popular understandings of HIV technologies and messages.

### The ways that people are socially organized and networked

Epidemiological categories (e.g., men who have sex with men, people from endemic countries, low-risk heterosexuals) have heuristic value as counting devices, but are inadequate proxies for the ways in which people do, in fact, interact with each other, and translate badly over the epistemic divide into everyday experience. How people are socially organized and networked is important for understanding the patterns of movement of HIV and also for the ways in which people can be reached or mobilized for prevention [57-60].

Molecular epidemiology has much to contribute to delineating the uneven bursts of HIV transmission that make up the larger epidemic [61,62], but there is much to be learned about how people on the leading edge of the epidemic are networked with each other, and the awareness they may have of their own sero-status and of those in their immediate social environments. Psychological research has identified a range of variables associated with unprotected anal intercourse and Ron Stall [63] and associates have adopted the term, “syndemic” to refer to this coincidence of epidemics of childhood sexual abuse, depression, partner violence and polydrug use. This and perhaps other syndemics have a social face as well -circuits, micro-cultures, social niches and social networks - and yet there is insufficient ethnography of these most vulnerable subsets of at-risk populations.

### The popular strategies and folk wisdoms that developed in the face of HIV risk

Counting risk “behaviour” widespread in the health sciences, tells us only so much [64]. Less is known about practices embedded in the exigencies and choices of everyday life, or the popular strategies and folk wisdoms for staying healthy [65,66]. Bio-technologies are also dependent on everyday practices; their use or disuse cannot simply be put down to “inadequate uptake” or a failure to be rational. The research question here is to investigate discourses available for making sense of risk. This means delving into, and working on, popular knowledge, moral reasoning and cultural presumptions that reduce (or enhance risk), and documenting narratives rooted in cultures of at-risk communities. This is not simply to affirm these practices and perceptions but to engage with them, work with them and develop knowledge grounded in them. There is evidence, for example, that some of the vulnerabilities to transmission occurring among gay and bisexual men stem *not* from inadequate knowledge or psychological deficiencies, but rather from inconsistent assumptions and interpretations of the “rules of the game” governing sexual interactions [58].

### Socio-historical movement of sexual and drug cultures

These are the master frameworks through which risk, values and, indeed, risks worth taking are assessed. The entire expensive, painstakingly evaluated edifice of intensive practitioner-delivered lifestyle-change interventions, with the *Effective Behavioral Intervention* imprimatur of the Centers for Disease Control and Prevention, rise or fall on this movement. The intervention that may be fascinating and fashionable at one moment can turn out to be stale and *passé* at another. These movements shape, as well, the experience of entire generations and intersect with the personal development of individuals in the moving cultures in which they participate [67]. To pick just one example, social research is only beginning to come to grips with the rapid virtualization of the sexuality of a wired generation that has ready access to imagery and internet networks well before embarking on practice.

### The dynamics of popular mobilization to advance health

Social movement analysis has rarely been applied to successful HIV health mobilization, but Toorjo Ghose [68] and colleagues point out its usefulness for Songachi. Though this framework is not often thought of in reference to drug users, they too can be understood as collective actors.

While injection drug users (IDUs) have clearly changed their behaviour to protect themselves from becoming infected with HIV, they have also dramatically changed their behaviour to protect their peers and sexual partners from becoming infected. IDUs have shown multiple altruistic responses to HIV/AIDS. The development of new social norms against sharing needles and syringes is one example. The effectiveness of HIV prevention for IDUs should not be viewed only in terms of programmes influencing individuals, but also more as a collective response by the IDU community to reduce HIV risk behaviour [60].

While lesbian, gay, bisexual and transgendered (LGBT) communities have been perhaps the textbook case of community mobilization, recent observers question if this is a historical moment that has passed as these communities appear to be fragmenting into smaller scenes and groups [69]. The political organization of LGBT communities has moved in a similar direction toward focused, diversified and multiple organizational nodes that are nevertheless still connected rhizomatically, that is, through often informal, *ad hoc*, and not readily visible networks [70]. This more decentralized, tribalized form of social connection is fundamental to understand as prevention work must adapt to the multiplicity of networks and their cultures.

There are several challenges that present themselves in understanding and engaging contemporary forms of community mobilization. The first is to delineate the smaller scenes, micro cultures, tribes and subsets of at-risk populations so that their discourses and concerns might be better addressed. In addition, there may still be potential in generating social forums for “communicative action” a significant vehicle for social change according to the leading social theorist, Jürgen Habermas [71], and one of the few available to HIV prevention. Both strategies could make good use of social media especially to engage a wired generation that is connected and accessible in new ways.

One major intervention of this kind is http://hivstigma.com, an innovative web-supported stigma-reduction intervention for gay and bisexual men, a project intended to open a forum to allow community members to advance a dialogue on community ethics with direct impact on practices related to HIV transmission [41]. Relying on traditional and new media of communication, hivstigma. com provided virtual space to develop community engagement with the question of HIV stigma. This intervention also raises the question of whether decentralizing trends among gay and bisexual men should be treated simply as a given. The creation of a communication centre, this time in cyberspace, revealed an appetite for community-wide dialogue and a willingness to engage a sense of collective fate that could be affected by the everyday practices of HIV-positive and HIV-negative men.

### The institutional sources of HIV discourses

Understanding risk perception necessitates research on institutional sources influencing both popular and policy orientations to the epidemic. Schools, mass media, churches and mosques, the judiciary, biomedicine and the Internet are all major actors in framing the meaning of HIV and the means for addressing HIV risk. Indeed they are actors with much more institutional solidity and pervasive influence than all of the community-based organizations and public health authorities devoted to HIV prevention. Just what kinds of messages flow from these institutions and the “semiotic snares” [72,73] they create in everyday practice are fundamental to making sense of how and why transmission occurs.

### Popular understandings of HIV technologies and messages

Just how the actuarial reasoning of health science translates into personal risk strategies requires investigation. HIV technologies and messages occur in a context of communication in relationships, workplace exigencies, and popular moral reasoning. Even the widespread claim that gay men have become complacent because of antiretrovirals is poorly documented. The HIV optimism hypothesis functions more as an observer’s rule [74], that is, an explanation that “makes sense” and circulates among scientists, than it does as a rule of thumb for gay and bisexual men themselves. Actual investigation of the views of gay and bisexual men assessing risk in their day-to-day interactions typically finds a much more complex array of considerations. HIV optimism carries very different meanings for HIV-positive and HIV-negative men and plays, at most, a minor role in risk situations [40,75]. Also, how the treatment-as-prevention mantra propounded by biomedical “experts” translates into everyday risk management is not well documented.

## Conclusions

This paper raises the question of how knowledge creation is organized in the area of HIV prevention and how this concatenation of expertise, resources, at-risk people and viruses shapes the knowledge used to impede the epidemic. Much of the organizational and investment centre point of HIV prevention appears to be occupied by a search for biomedical technologies, and perhaps more importantly by an epistemological frame characteristic of biomedical individualism [72,76]. This frame largely bypasses the social, or assigns it to categories of “inadequate uptake” patient management or the residual category of the inexplicable. When the social is brought into the frame, it is very often in the form of “experimental manipulations [that] remove the very stuff that produces change - the social glue that makes us social beings” [17].

At its best, HIV prevention studies could look toward ways in which biomedical and social approaches to HIV prevention would work synergistically [77] by moving past the techno-eschatology that currently characterizes much of the field and working seriously with the social and community resources already at hand.

## Competing interests

The author has no competing interests to declare.

## Author’s information

BDA is University Professor of Sociology at the University of Windsor and Senior Scientist and Director of Prevention Research at the Ontario HIV Treatment Network. He is(co-)author of four books and numerous articles on HIV prevention and social aspects of HIV.
